# Iron Single Atoms Anchored on Nitrogen-Doped Carbon Matrix/Nanotube Hybrid Supports for Excellent Oxygen Reduction Properties

**DOI:** 10.3390/nano12091593

**Published:** 2022-05-07

**Authors:** Yining Jia, Chunjing Shi, Wei Zhang, Wei Xia, Ming Hu, Rong Huang, Ruijuan Qi

**Affiliations:** 1 Key Laboratory of Polar Materials and Devices (MOE), Department of Electronics Sciences, School of Physics and Electronic Science, East China Normal University, Shanghai 200062, China; 51191213010@stu.ecnu.edu.cn (Y.J.); chunjingshi620@163.com (C.S.); wzhang@ee.ecnu.edu.cn (W.Z.); mhu@phy.ecnu.edu.cn (M.H.); 2 Shanghai Key Laboratory of Green Chemistry and Chemical Processes, School of Chemistry and Molecular Engineering, East China Normal University, Shanghai 200241, China; xiaweifriend@163.com; 3 Collaborative Innovation Center of Extreme Optics, Shanxi University, Taiyuan 030006, China

**Keywords:** oxygen reduction reaction, electrocatalyst, single atom catalysts, carbon nanotubes

## Abstract

Single-atom non-precious metal oxygen reduction reaction (ORR) catalysts have attracted much attention due to their low cost, high selectivity, and high activity. Herein, we successfully prepared iron single atoms anchored on nitrogen-doped carbon matrix/nanotube hybrid supports (FeSA-NC/CNTs) by the pyrolysis of Fe-doped zeolitic imidazolate frameworks. The nitrogen-doped carbon matrix/carbon nanotube hybrid supports exhibit a specific surface area of 1626.814 m^2^ g^−1^, which may facilitate electron transfer and oxygen mass transport within the catalyst and be beneficial to ORR performance. Further electrochemical results revealed that our FeSA-NC/CNTs catalyst exhibited excellent ORR activity (half-wave potential: 0.86 V; kinetic current density: 39.3 mA cm^−2^ at 0.8 V), superior to that of commercial Pt/C catalyst (half-wave potential: 0.846 V; kinetic current density: 14.4 mA cm^−2^ at 0.8 V). It also has a great stability, which makes it possible to be a valuable non-noble metal electrode material that may replace the latest commercial Pt/C catalyst in the future.

## 1. Introduction

To accelerate the large-scale commercialization of metal–air battery technologies, low-cost, high activity platinum group metal-free (PGM-free) catalysts for oxygen reduction reactions (ORR) have been developed as alternatives for the scarce and high-cost Pt-based catalysts [1,2]. In this context, single-atom metal–N–C catalysts (SACs) have consequently attracted significant research interest for their unique electronic and geometric structures, permitting the maximum atom-utilization efficiency. The metal atom center in such metal–N–C catalysts should not be considered as an isolated active site, which is coordinated with the surrounding carbon substrate structure and acts as an integral part of the catalytic reaction [3]. Among all the PGM-free ORR catalysts, Fe–N–C has attracted much interest due to its brilliant performance and stability [4,5]. Fe–N_4_ moieties, with the geometric structure of the Fe atom coordinated with four nitrogen atoms, are widely regarded as the active sites to directly adsorb O_2_ and catalyze the subsequent O–O bond breaking [6,7], therefore significantly improving the ORR catalytic activity [8,9]. Thus, the overall activity of Fe–N–C atomic catalysts is related to two key factors: the formation of Fe–N_4_ moieties as well as the structural morphology of the supports. At present, although some success has been achieved in exposing more active sites to promote catalytic performance [10,11], the role of the N defect site configuration in Fe–N–C electrocatalysts is still difficult to determine. To this end, metal–organic frameworks (MOFs) are widely used as precursors in research due to their homogeneous chemical composition and abundant microporous structure where Fe–N_x_ moieties can largely be hosted [12,13,14]. To enhance the widespread exposure of active sites hidden inside the sample, many strategies have been used to tailor the morphology and nanostructure of Fe–N–C electrocatalysts [15,16] in which the formation of N-doped carbon (NC)/carbon nanotube (CNT) hybrid supports by MOF pyrolysis with the presence of Fe, Co, or other metals are widely reported [17,18]. The catalyst supports consisting of highly graphitized carbon or CNTs are more stable and possess high conductivity, which are beneficial to high electrocatalytic performance in ORR [19]. However, as reported, metal nanoparticles (NPs) are easily formed at the end of the CNTs, which in turn reduces the utilization of active sites [20,21]. The presence of CNTs and the absence of metal NPs require a delicate kinetic balance. As far as we know, iron single atoms (SAs) anchored on both CNTs and the carbon matrix from MOF pyrolysis have seldomly been reported.

In this work, we successfully prepared iron single atoms anchored on nitrogen-doped carbon/carbon nanotube (FeSA-NC/CNTs) hybrid supports by the pyrolysis of ZIF-8 as molecular cages in one step without any further treatment. The aberration-corrected high-angle annular dark field scanning transmission electron microscopy (AC-HAADF-STEM) characterization showed that abundant Fe atoms were uniformly distributed on the NC/CNT supports without any Fe NPs being observed. Electron energy-loss spectroscopy (EELS) spectra showed the existence of Fe and N in the same area. Further extended X-ray absorption fine structure (EXAFS) indicated that four nitrogen atoms were coordinated around each Fe atom to form a stable Fe–N_4_ structure. The tailored porous carbon matrix/nanotube structures are beneficial to the full utilization of Fe–N_4_ sites [22], which makes our FeSA-NC/CNTs catalyst deliver not only better ORR performance, but also more excellent stability than that of the commercial Pt/C catalyst (20 wt%) under alkaline conditions. Our study provides a new idea for the design and synthesis of efficient single-atom non-precious metal catalysts, providing an important reference for the development of new high-efficiency electrocatalysts.

## 2. Experimental Section

### 2.1. Reagents

2-Methylimidazole (98%, Aladdin, Shanghai, China), zinc nitrate hexahydrate (analytical grade, 99%, Aladdin), iron acetylacetonate (98%, Aladdin), methanol (analytical grade, Sinopharm Chemical, Shanghai, China), commercial Pt/C (20 wt% metal, Alfa Aesar, Shanghai, China), KOH (analytical grade, Sinopharm Chemical), and Nafion D-521 dispersion (5% *w*/*w* in water and 1-propanol, Alfa Aesar) were used as received without any further purification. The distilled water used in all experiments was obtained through ion-exchange and filtration.

### 2.2. Synthesis of ZIF-8

In the typical synthesis of ZIF-8, Zn(NO_3_)_2_·6H_2_O (0.6082 g, 2 mmol) was dissolved in 15 mL methanol with stirring for 15 min in beaker A. 2-Methylimidazole (1.0604 g, 13 mmol) was dissolved in 7.5 mL methanol with stirring for 15 min in beaker B. Then, the solution in beaker B was subsequently added into beaker A with thorough stirring for 0.5 h at room temperature. The mixed solution was then transferred into a 45 mL Teflon lined stainless-steel autoclave and kept at 90 °C for 1 h in an oven. After cooling to room temperature, the obtained product was separated by centrifugation and washed with anhydrous ethanol three times and finally dried overnight under vacuum at 60 °C.

### 2.3. Synthesis of Fe(acac)_3_-0.1@ZIF-8

The synthesis process is the same as ZIF-8, with 71.6 mg (0.2 mmol) Fe(acac)_3_ being introduced.

### 2.4. Synthesis of Fe(acac)_3_-0.15@ZIF-8

The synthesis process was the same as ZIF-8, with 107.4 mg (0.3 mmol) Fe(acac)_3_ being introduced.

### 2.5. Synthesis of NC, FeSA-NC/CNTs and FeNP-NC/CNTs

The powders of ZIF-8 were transferred into a ceramic boat and placed in a quartz tube furnace. Then, the sample was heated to 900 °C at a heating rate of 5 °C min^−1^, kept at 900 °C under flowing N_2_ for 3 h and finally naturally cooled to room temperature. The final powders NC were collected and characterized without further treatment.

The synthesis process of FeSA-NC/CNTs was the same as NC, except that the raw material was Fe(acac)_3_-0.1@ZIF-8.

The synthesis process of FeNP-NC/CNTs was the same as NC, except that the raw material was Fe(acac)_3_-0.15@ZIF-8.

### 2.6. Synthesis of FeSA-NC

The powders of Fe(acac)_3_-0.1@ZIF-8 were transferred into a ceramic boat and placed in a quartz tube furnace. Then, the sample was heated to 800 °C at a heating rate of 5 °C min^−1^, kept at 800 °C under flowing N_2_ for 3 h, and finally naturally cooled to room temperature. The final powders of FeSA-NC were collected and characterized without further treatment.

### 2.7. Characterization

The morphology of the samples was characterized by scanning electron microscopy (SEM, Gemini 450, ZEISS, Jena, Germany) with an acceleration voltage of 5 kV. The transmission electron microscopy (TEM) images and element mappings were obtained at 200 kV using a JEM-2100F (JEOL, Tokyo, Japan) equipped with an X-ray energy dispersive spectrometer (EDS: X-Max 80T, Oxford, UK) for chemical composition analysis. EDS elemental maps were taken in HAADF-STEM mode. Atomic resolution analyses were performed on an aberration-corrected scanning transmission electron microscopy (AC-STEM, Grand ARM300F, JEOL, Japan) equipped with an electron energy-loss spectrometer (EELS: GIF Quantum 970, Gatan, Inc., CA, USA). The energy resolution of EELS was ∼1 eV measured at the width at half-maximum of the zero-loss peak with the energy dispersion of 0.25 eV/channel. The structures were characterized by X-ray diffraction (XRD, PANalytical Empyrean Rayon X, Eindhoven, The Netherlands) with Cu Kα radiation (λ = 1.5418 Å) at 40 kV and 40 mA with an increment of 0.04 degrees. Raman spectra were collected on a Renishaw inVia confocal Raman microscope with a 532 nm wavelength incident laser light. X-ray photoelectron spectroscopy (XPS, Kratos, AXIS Ultra DLD, Manchester, UK) was performed to investigate the chemical bond using Al K Alpha (1486.6 eV) and the value of 284.8 eV as the C 1s peak reference. A PerkinElmer Pyris Diamond was utilized for TGA measurements. The differential scanning calorimetry (DSC) was recorded on a Diamond DSC system. Nitrogen adsorption–desorption measurements were conducted on an Autosorb IQ Gas Sorption System at 77 K. The Brunauer–Emmett–Teller (BET) surface area was calculated using the adsorption data. X-ray absorption fine structure (XAFS) measurements based on Synchrotron Radiation were carried out at the 1W2B beamline at the Beijing Synchrotron Radiation Facility (BSRF), China. The EXAFS data were processed according to the standard procedures using the ATHENA module implemented in the IFEFFIT software packages (version 0.9.25, Chicago, IL, USA).

### 2.8. Electrochemical Measurements

A total of 2 mg of catalyst was dispersed in 1 mL Nafion (5 wt%) and sonicated for about an hour under ultrasonic treatment to form a homogeneous catalyst ink. A sample of 24.7 μL ink was dropped onto the polished glassy carbon disk electrode in order to yield a catalyst loading of 0.2 mg cm^−2^ and dried under an infrared lamp.

The electrochemical impedance spectroscopy (EIS) and all the electrocatalytic performance measurements were performed using a CHI 760E Electrochemical Workstation (Shanghai Chenhua, Shanghai, China). The EIS curves were obtained at the reduced peak potential with a signal amplitude of 5 mV s^−1^ and a frequency range of 100 kHz–100 mHz.

The oxygen reduction performance was conducted in a three-electrode system: a platinum foil as counter electrode; a saturated calomel electrode (SCE) as the reference electrode, and a glassy carbon electrode as the working electrode. All potential values were calibrated to the reversible hydrogen potential (E_RHE_) based on the Nernst equation:

(1) $$E_{\mathrm{RHE}}=E_{\mathrm{SCE}}+0.2415+0.0591*\mathrm{pH}$$

O_2_ and N_2_ were saturated in 0.1 M KOH, respectively, and used as an electrolyte at room temperature. Cyclic voltammetry (CV) experiments were conducted with a sweep rate of 50 mV s^−1^ in the potential ranging from 0.1 to 1.2 V in O_2_-saturated electrolyte at a rotating disk electrode (RRDE) of 0 rpm. Then linear sweep voltammetry (LSV) experiments were carried out in the potential range from 0.1 to 1.2 V at a rotating speed ranging from 400 to 2025 rpm with a sweep rate of 5 mV s^−1^ at room temperature. The accelerated durability tests (ADT) of the electrocatalyst were acquired in an O_2_-saturated 0.1 M KOH electrolyte at room temperature, with potential cycling between 0.6 to 1 V at a sweep rate of 50 mV s^−1^ for 5000 cycles.

The electronic transfer number (n) was analyzed by the Koutecky–Levich (K–L) equation:

(2) $$\frac{1}{J}=\frac{1}{J_{L}}+\frac{1}{J_{K}}=\frac{1}{{B\omega }^{\frac{1}{2}}}+\frac{1}{J_{K}}$$

(3) $$B=0.62{\mathrm{nFC}}_{0}D_{0}^{\frac{2}{3}}V^{-\frac{1}{6}}$$

where J represents the current density; $J_{L}$ and $J_{K}$ are the limited and kinetic current density, respectively; ω indicates the rotating rate of the electrode; n is the electron transfer number in oxygen reduction; F is the Faraday constant (96,485 C mol^−1^); $C_{0}$ is the bulk concentration of O_2_ (1.2 × 10^−6^ mol cm^−3^); $D_{0}$ is the diffusion coefficient of O_2_ in 0.1 M KOH (1.9 × 10^−5^ cm^2^ s^−1^); V is the kinematic viscosity of the electrolyte (0.01 cm^2^ s^−1^); and the constant 0.62 was used to determine B when the unit of rotating rate was rad s^−1^.

The H_2_O_2_ yield (H_2_O_2_%) and the electron transfer number (n) were calculated with the following equations:

(4) $$H_{2}O_{2}\;\left(\%\right)=200\times \frac{\frac{I_{R}}{N}}{I_{D}+\frac{I_{R}}{N}}$$

(5) $$n=4\times \frac{I_{D}}{I_{D}+\frac{I_{R}}{N}}$$

where $I_{D}$ is the disk current; $I_{R}$ is the ring current; and N is the ring collection efficiency with a value of 0.4.

The Tafel plot was calculated with the following equation:

(6) $$\eta =a+b\;\log\left|J_{K}\right|$$

where η is overpotential; $J_{K}$ means kinetic current density; and b indicates the Tafel plot. According to Equation (6), to make the $\eta -\log\left|J_{K}\right|$ curve, take the part that fits the linear relationship, and its slope is the Tafel plot.

## 3. Results and Discussion

**Figure 1:**
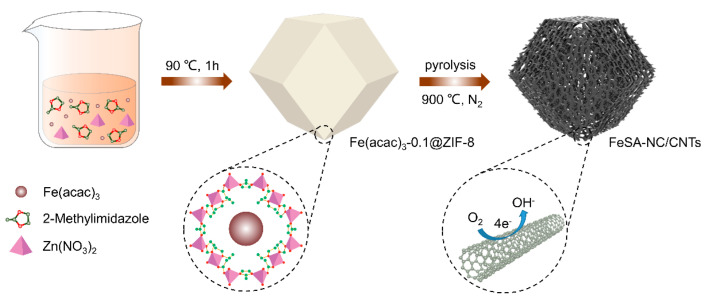
The schematic illustration of FeSA-NC/CNTs.

The synthesis process of Fe(acac)_3_-0.1@ZIF-8 and its subsequent conversion into FeSA-NC/CNTs is schematically illustrated in Figure 1. Fe(acac)_3_ was mixed with ZIF-8 according to the synthesis method in the previous study [23]. A molecular-scale cage was formed by Zn^2+^ and 2-methylimidazole with the poles and cavities being larger for one Fe(acac)_3_ molecule to be trapped. After pyrolysis at 900 °C under N_2_ flow, the final product, FeSA-NC/CNTs, was obtained after zinc evaporation at a temperature higher than 450 °C (see Figure S1 for the DSC/TGA results) [24]. The target metal (Fe) sites were spatially separated by the 2-methylimidazole bond and zinc atoms, with a greatly increasing space distance between one other. Therefore, ZIF-8 can be transformed into nitrogen-doped carbon (NC)/carbon nanotube (CNT) hybrid supports after the evaporation of zinc atoms during high temperature heat treatment in N_2_. A suitable amount of nitrogen dopant in carbon can effectively maintain good electrical conductivity while improving its electrocatalytic properties. Meantime, an appropriate content of Fe(acac)_3_ could be carbonized by the organic ligands to form isolated single iron atoms bound to the nitrogen species anchored on the carbon matrix/CNTs [25].

**Figure 2:**
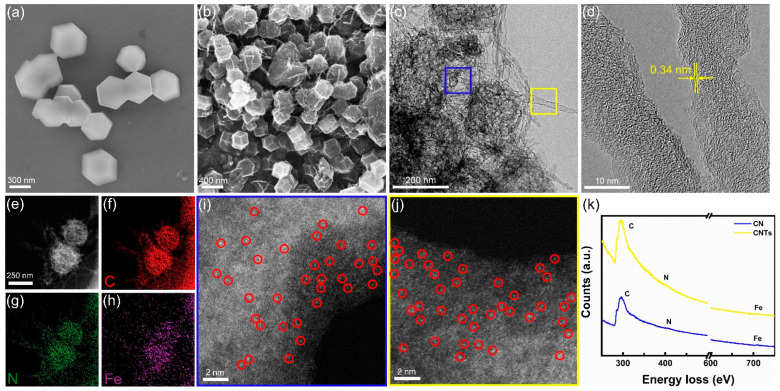
SEM images of (**a**) Fe(acac)_3_-0.1@ZIF-8 and (**b**) FeSA-NC/CNTs. (**c**) TEM and (**d**) HRTEM images of FeSA-NC/CNTs. (**e**–**h**) STEM-EDS elemental maps of C, Fe, and N of the FeSA-NC/CNTs sample shown in (**e**). AC-HAADF-STEM images of (**i**) N-doped carbon matrix and (**j**) carbon nanotubes, corresponding to the blue and yellow areas in (**c**), respectively. (**k**) Electron energy-loss spectroscopy showing the C K-edge, N K-edge, and Fe L-edge acquired from the marked region in (**c**).

The morphologies of the original Fe(acac)_3_-0.1@ZIF-8 and as-prepared FeSA-NC/CNTs were examined by using SEM and TEM. As shown in Figure 2a and Figure S2, the original Fe(acac)_3_-0.1@ZIF-8 exhibited rhombic dodecahedral morphology with a particle size of about 400 nm [24]. After pyrolysis at 900 °C under N_2_ for 3 h, the sample still maintained its previous rhombic dodecahedral morphology with many thread-like or filamentous substances decorated on the surface of the particles (Figure 2b). The TEM and HRTEM images in Figure 2c,d show that these crisscross substances were thin-walled carbon nanotubes with a diameter of around 10 nm. No obvious nanoparticles (NPs) were observed on these carbon nanotubes or the rhombododecahedral carbon matrix, implying the possible formation of Fe single atoms. Corresponding STEM-EDS elemental maps in Figure 2e–h demonstrate that the signals of Fe, N, and C were uniformly dispersed on the NC and CNTs.

Furthermore, AC-HAADF-STEM was used to visualize the microstructure of NC and CNTs at the atomic scale. Due to the much higher atomic number (Z) of the Fe atoms compared to that of C and N atoms [26], bright dots representing Fe single atoms distributed on NC as well as CNTs can be observed in HAADF images (highlighted by red circles in Figure 2i–j and Figure S3). Moreover, EELS spectra taken at the carbon matrix and carbon nanotubes (Figure 2k) indicated the co-existence of Fe, N, and C, demonstrating the formation of abundant Fe–N_4_ moieties. As known, for SACs, if metallic catalysts are not completely encapsulated or simply anchored on the surface of the carbon-based support, they are not effective at preventing the leaching of metallic ion under harsh operating conditions. In addition, fully encapsulating the metal catalysts into a thicker carbon matrix, but away from the surface, may hinder the effective electron transfer between the catalysts and the reactants [27]. In our work, the NC/CNT hybrid supports possessed much higher surface area (1626.814 m^2^ g^−1^) thanks to their mesoporous structure (Figure S4), which is not only beneficial to the full utilization of Fe–N_4_ active sites [28], but may also facilitate oxygen mass transfer within the catalyst film. Therefore, our FeSA-NC/CNTs catalyst exhibited a high electrocatalytic activity and stability in ORR [19]. These aspects demonstrate the advantages of manufacturing well-dispersed Fe–N–C catalysts by using MOFs as a template.

To further explore the formation mechanism of FeSA-NC/CNTs, MOF precursors with different contents of Fe(acac)_3_ were synthesized. It was found that after pyrolysis at the same conditions, FeNP-NC/CNTs with obvious Fe NPs could be observed at the end of the formed CNTs (Figure S5) when the content of Fe(acac)_3_ in ZIF-8 was changed from 0.1 to 0.15. Moreover, when the pyrolysis temperature was reduced to 800 °C, only Fe single atoms (SAs) anchored on NC were formed while neither Fe NPs nor CNTs were formed (Figure S6). Based on the above results, it is speculated that the formation of Fe SAs on both NC and CNTs can be attributed to delicately tuning the content of Fe(acac)_3_ in ZIF-8 and pyrolysis temperature. The content of doped Fe(acac)_3_ should be controlled, as the ORR performance is far from being ideal if the amount is small (less active sites are involved) and clusters or eventually nanoparticles will be formed if the amount is too large [29,30]. Besides, higher pyrolysis temperature will be critical for improving the graphitization of carbon to form a porous carbon matrix as well as CNTs with the existence of Fe.

**Figure 3:**
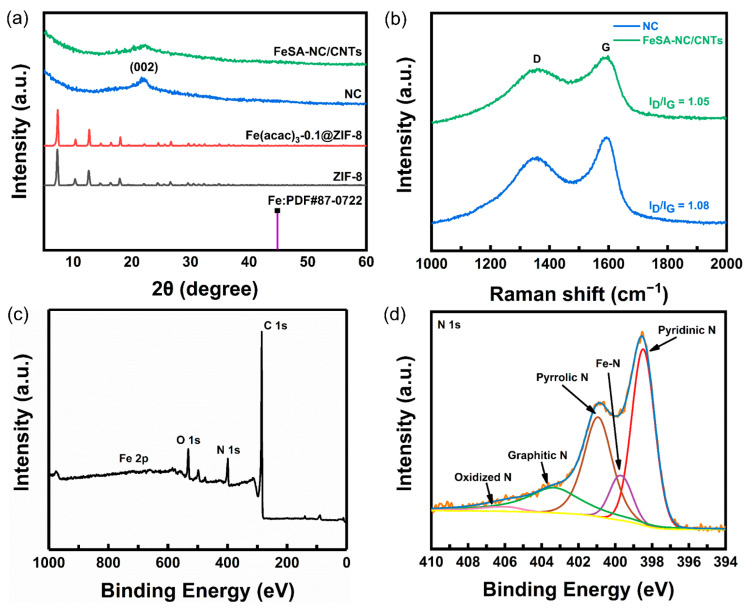
(**a**) XRD patterns of ZIF precursors and as-pyrolyzed samples. (**b**) Raman spectra. (**c**) XPS survey scan spectrum of FeSA-NC/CNTs. (**d**) High resolution XPS N 1s spectra of FeSA-NC/CNTs.

XRD, Raman, and XPS were employed to further investigate the structural properties and chemical composition of the electrocatalysts. The XRD patterns of original ZIF-8 and Fe(acac)_3_-0.1@ZIF-8 powders (Figure 3a) showed almost identical diffraction peaks, indicating that the addition of Fe(acac)_3_ does not change the structure of ZIF-8 [31,32]. Similar to the XRD pattern of NC derived from pure ZIF-8 (the blue line in Figure 3a), there was a much broader diffraction peak in the range of 20–30° (indexed to (002) planes of graphitic carbon) for that of FeSA-NC/CNTs (the green line in Figure 3a), demonstrating the formation of a graphitic carbon structure [33], whereas the degree of graphitization was not good enough. Furthermore, no diffraction peak corresponding to the bulk-centered cubic α-Fe (PDF#87-0722) was detected in Figure 3a, which proves that there was no formation of any iron nanoparticles in FeSA-NC/CNTs. In contrast, the XRD pattern of FeNP-NC/CNTs (Figure S7) showed a small but distinct diffraction peak at about 44°, which verified the presence of Fe NPs. As seen in the Raman spectra shown in Figure 3b, the peaks of the D-band and G-band for both the NC and FeSA-NC/CNTs were roughly at 1354 cm^−1^ and 1591 cm^−1^, respectively. It is well-known that the ratio of the integrated area of the D-band to the G-band is related to the defect density in the lattice [33]. The decrease in this ratio from NC to FeSA-NC/CNTs implies that the doping of iron drives an increase in the graphitization of ZIF-8-derived carbon hybrid supports, in line with the XRD results, which may improve the electrical conductivity of the carbon supports and promote electron transfer for electrochemical applications [34]. The XPS spectra of FeSA-NC/CNTs (Figure 3c) showed four peaks for C 1s, N 1s, O 1s, and Fe 2p, confirming the presence of carbon, nitrogen, oxygen, and especially iron, which is present in a small but real amount. It should be noted that the O 1s peak may come from surface adsorbed oxygen in the environment. The C 1s peak (Figure S8a) can be deconvoluted into three peaks centered at 284.8, 286.0, and 290.0 eV, which can be attributed to the C–C, C–N, and C=O bonds, respectively [35]. The XPS spectrum of Fe 2p showed two spin-orbit doublets at 711.7 and 722.9 eV, which can be attributed to the Fe 2p_3/2_ and Fe 2p_1/2_ orbits. Unfortunately, the Fe signal was not very visible, perhaps related to the coverage of carbon matrix/carbon nanotubes and/or the iron content below the detection limit (Figure S8b). The presence of C–N bonds indicates the successful nitrogen doping in the carbon matrix. In addition, high-resolution N 1s spectra showed the presence of porphyrin-like Fe–N_4_ moieties at 399.7 eV as well as pyridinic (398.5 eV), pyrrolic (400.9 eV), graphitic (403.5 eV), and N–O_x_ (406.3 eV) (Figure 3d) [36]. According to previous studies, pyridinic N and pyrrolic N can provide coordination sites by moving lone pairs to the carbon plane, which can enhance the chemisorption of oxygen molecules and intermediates during the ORR process [37,38]. Graphitic N not only improves the conductivity of the catalyst, but also the presence of C–N bonds can induce an inhomogeneous distribution of electrons, thus promoting the adsorption of O_2_ and the dissociation or weakening of O=O bonds [39,40,41].

**Figure 4:**
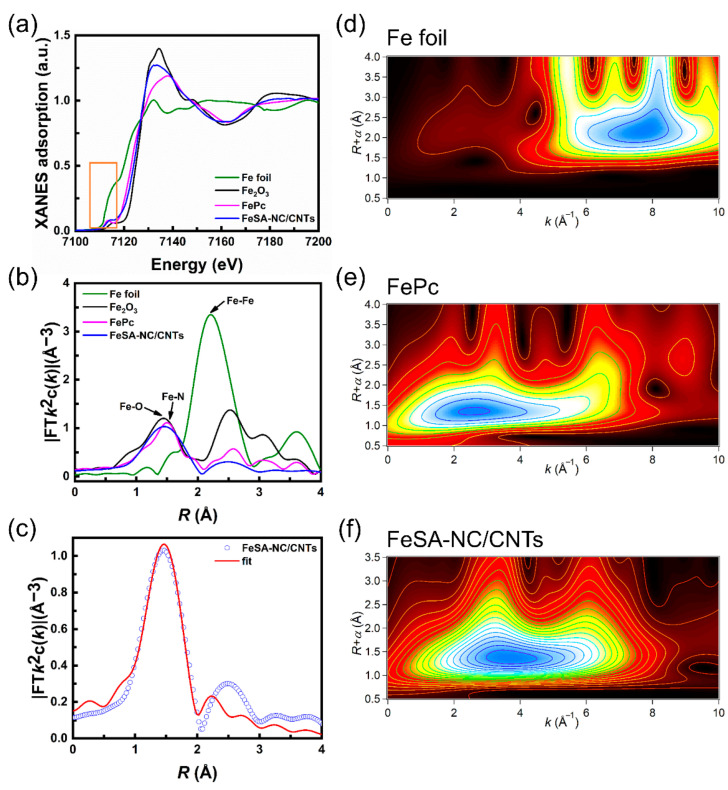
(**a**) Fe K-edge XANES spectra of FeSA-NC/CNTs (the orange area highlights the near-edge absorption energy). (**b**) Fourier transform (FT) of the Fe K-edge EXAFS spectra. (**c**) The corresponding EXAFS r space fitting curves of FeSA-NC/CNTs. Wavelet transform (WT) of Fe K-edge for (**d**) Fe foil, (**e**) FePc, and (**f**) FeSA-NC/CNTs.

To further investigate the chemical state and coordination environment of the Fe sites in FeSA-NC/CNTs, X-ray absorption near-edge structure (XANES) and extended X-ray absorption fine structure (EXAFS) were carried out at the Fe K-edge. Fe foil, FePc, and Fe_2_O_3_ samples were also measured as reference samples. As illustrated in the Fe K-edge XANES spectra of FeSA-NC/CNTs (Figure 4a), the adsorption edge was located between Fe foil and Fe_2_O_3_, indicating that the oxidation state of the Fe species was between Fe^0^ and Fe^3+^, and close to Fe^2+^. The EXAFS spectra of FeSA-NC/CNTs (Figure 4b) showed a prominent peak centered at 1.5 Å, which was mainly attributed to the first Fe–N coordination shell [42,43]. Moreover, compared to the Fe foil, the absence of scattering peaks derived from Fe–Fe coordination in FeSA-NC/CNTs suggests that Fe species were monodispersed in the N-doped carbon matrix/CNT hybrid supports [44]. The coordination structure of the Fe atoms in the FeSA-NC/CNTs was further investigated by quantitative EXAFS curve fitting analyses (Figure 4c and Table S1), which clearly revealed that the Fe center was coordinated with four N atoms at the first coordination shell. All fitting results were well consistent with the experimental data, from which the average coordination numbers of Fe–N was obtained as 4.2, with the average bond lengths for Fe–N of 2.01 Å, respectively (see more details in Table S2). Furthermore, the wavelet transform (WT, Figure 4d–f) results displayed only one WT intensity maximum at ≈3.8 Å^−1^, associated with the Fe–N pair. Compared with the WT plots of Fe foil, the WT signal related to Fe–Fe contribution was not detected in the FeSA-NC/CNTs. These observations further demonstrate that the single Fe atoms simultaneously coordinated with N atoms, forming the Fe–N bonds.

**Figure 5:**
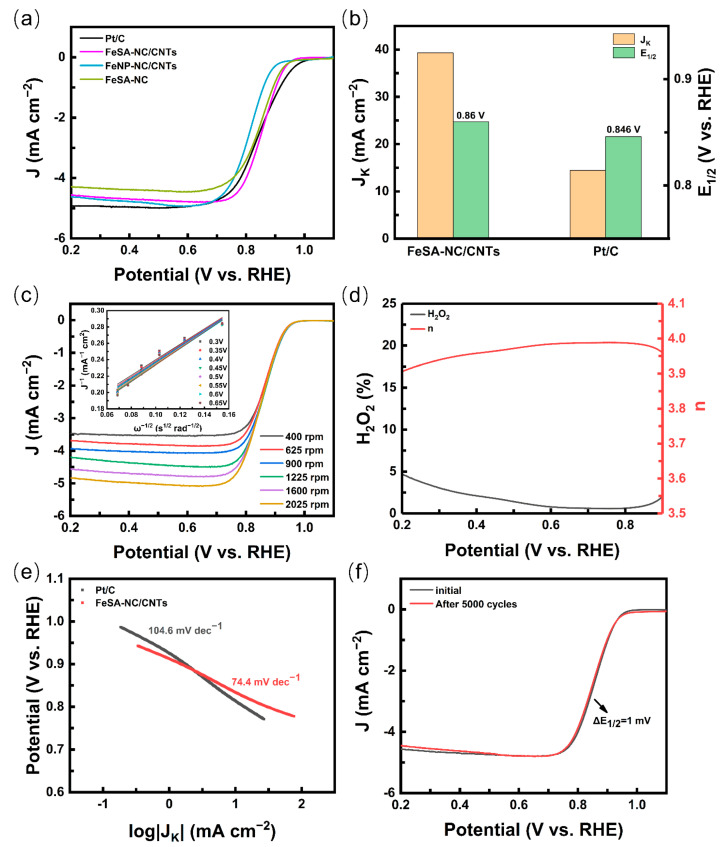
(**a**) ORR polarization plots of FeSA-NC/CNTs and Pt/C in O_2_-saturated 0.1 M KOH with a sweep rate of 5 mV s^−1^ and 1600 rpm. (**b**) E_1/2_ and J_K_ at 0.8 V for different catalysts. (**c**) LSV curves of FeSA-NC/CNTs with various rotation rates (inset: K–L plots). (**d**) Electron transfer number and H_2_O_2_ yield in ORR on FeSA-NC/CNTs from the RRDE results. (**e**) Tafel plots of Pt/C and FeSA-NC/CNTs. (**f**) LSV curves of FeSA-NC/CNTs before and after 5000 potential cycles.

The construction of the FeSA-NC/CNTs catalyst was dedicated to improving the ORR catalytic performance, which was investigated by cyclic voltammetry (CV, Figure S9) and linear scanning voltammetry (LSV) in an O_2_-saturated 0.1 M KOH solution based on a three-electrode system under rotating disk electrode (RDE). A commercial 20 wt% Pt/C catalyst (20 wt% metal, Alfa Aesar) was used as the reference for the performance comparison. All given potentials refer to the reversible hydrogen electrode (RHE). This ORR performance was compared in this work to other similar published research (Table S3). FeSA-NC/CNTs showed a reduction peak at 0.82 V, indicating a certain ORR activity (Figure S9). As shown in Figure 5a, the onset potential (E_onset_) with a value of about 0.93 V for FeSA-NC/CNTs was slightly inferior to that of Pt/C, but much better than that of FeNP-NC/CNTs and FeSA-NC. It demonstrates that the high-performance Fe-SAC catalyst can be prepared by tuning the content of Fe(acac)_3_ in ZIF-8 and the pyrolytic conditions. According to Figure 5b, FeSA-NC/CNTs deliver a higher half-wave potential (E_1/2_ = 0.86 V) than that of Pt/C (0.846 V). The kinetic current density (J_k_) of FeSA-NC/CNTs (39.3 mA cm^−2^) at 0.8 V was also much higher than that of Pt/C (14.4 mA cm^−2^), indicating its superior kinetics. To further unveil the electron transfer mechanism of FeSA-NC/CNTs, the LSV curves at different rotating rates are depicted in Figure 5c. The Koutecky–Levich (K–L) curves obtained from the LSV curves exhibited good linearity, showing the primary reaction kinetics related to the O_2_ concentration and the potential-independent electron transfer rate (Figure 5c, inset) [45]. The yield of H_2_O_2_ and electron transfer number were confirmed by the rotating ring-disk electrode (RRDE), suggesting a superior selectivity of oxygen reduction for H_2_O with an electron transfer number greater than 3.9 and a H_2_O_2_ yield below 5% over the potential range of 0.2 to 0.9 V (Figure 5d) [46]. The Tafel slope of FeSA-NC/CNTs was about 74.4 mV dec^−1^, which was significantly lower than that of Pt/C (104.6 mV dec^−1^), demonstrating the FeSA-NC/CNTs electrocatalyst possessed accelerated ORR kinetics (Figure 5e) [47]. It should be noted that there was no significant decay of E_1/2_ (ca. 1 mV) after 5000 consecutive potential cycles (Figure 5f), which proves that the FeSA-NC/CNT catalyst also has superb durability.

The electrochemical impedance spectroscopy (EIS) was also measured (Figure S10). The Nyquist plots were fitted by an equivalent circuit model. As shown in Figure S10a–c, FeSA-NC/CNTs showed the smallest arc radius (R_ct_: 100.3 Ω) in the Nyquist plot compared with FeNP-NC/CNTs and FeSA-NC, indicating the lowest charge-transfer resistance at the catalyst/electrolyte interface and superior charge transport kinetics [48]. The Bode plots can be used to estimate the effectiveness of ion diffusion. The ion diffusion in the low-frequency region is related to the phase angle. The smaller the phase angle, the faster the ion diffusion [49]. Therefore, FeNP-NC/CNTs have a more favorable diffusion angle (<−40°). The phase angle of FeSA-NC/CNTs and FeSA-NC was close to 0° in the low-frequency region, demonstrating that diffusion was not the dominant mechanism. At lower frequencies, the impedance (|Z|) was much higher due to mass transfer effects, while at higher frequencies, the lower the impedance, the easier the charge transfer. FeSA-NC/CNTs had the lowest impedance at high frequencies (65 Ω), implying that charge transfer dominates. It can be seen that the value of |Z| decreased the most in FeNP-NC/CNTs, implying the breaking of the activation barrier and promoting charge transfer in the high-frequency region and charge diffusion in the low-frequency region.

## 4. Conclusions

In summary, we synthesized a single-atom FeSA-NC/CNTs catalyst through the cage-encapsulated-precursor pyrolysis strategy by skillfully tuning the pyrolyzed temperature and the content of Fe(acac)_3_ in the precursors. Based on the results of HRTEM, EDS, and HAADF-STEM, it is clear that the catalyst has a large number of Fe single atoms distributed on the NC/CNT hybrid supports as a mesoporous structure with a specific surface area of 1626.814 m^2^ g^−1^. Benefiting from the high density and superb accessibility of Fe–N_4_ active sites, the catalyst exhibited excellent ORR performance with a half-wave potential (E_1/2_) of 0.86 V, which exceeded that of commercial Pt/C. It had a high kinetic current density (J_k_) of 39.3 mV cm^−2^ at 0.8 V. The yield of H_2_O_2_ was below 5% and the electron transfer number was close to 4, indicating that FeSA-NC/CNTs had a high electrocatalytic efficiency. Besides, it showed excellent stability with little change in the ORR polarization curve after 5000 CV cycles. In short, the outstanding catalytic activity of FeSA-NC/CNTs can be attributed to the synergistic roles of abundant Fe single atoms as well as the porous NC/CNT hybrid architectures, which ensures the full utilization of Fe–N_4_ active sites with improved electron transfer and oxygen mass transport.

## Data Availability

The data presented in this study are available on request from the corresponding author.

## Supplementary Materials

The following supporting information can be downloaded at: https://www.mdpi.com/article/10.3390/nano12091593/s1, Figure S1: (a) TGA and (b) DSC curves of Fe(acac)_3_-0.1@ZIF-8. The weight loss was attributed to the decomposition of ZIF-8 and the release of Zn species. That is, the Zn nodes with a low boiling point of 907 °C would evaporate at such high temperatures, leaving the N rich defects; Figure S2: TEM image of Fe(acac)_3_-0.1@ZIF-8; Figure S3: (a) and (b) are representative HAADF-STEM images of FeSA-NC/CNTs at different areas; Figure S4: (a) N_2_ sorption isotherms of FeSA-NC/CNTs. (b) Corresponding pore size distribution curve calculated using the DFT methods; Figure S5: (a) TEM and (b) HAADF-STEM images of FeNP-NC/CNTs, where typical iron single atoms are marked by red circles; Figure S6: (a) TEM and (b) HAADF-STEM images of FeSA-NC, where typical iron single atoms are marked by red circles; Figure S7: XRD patterns of FeSA-NC/CNTs and FeNP-NC/CNTs; Figure S8: (a) High resolution XPS C 1s spectra of FeSA-NC/CNTs. (b) High resolution XPS Fe 2p spectra of FeSA-NC/CNTs.; Figure S9: CV curves of FeSA-NC/CNTs in O_2_-saturated 0.1 M KOH with a sweep rate of 50 mV/s; Figure S10: Nyquist plots of (a) FeSA-NC/CNTs, (b) FeSA-NC, and (c) FeNP-NC/CNTs, where the inset is the equivalent circuit model for impedance spectra fitting. Bode plots of (d) FeSA-NC/CNTs, (e) FeSA-NC, and (f) FeNP-NC/CNTs. Table S1: Fitting results of Fe foil EXAFS; Table S2: Fitting results of sample–Fe EXAFS; Table S3: Comparison of ORR performance with some reported non-precious catalysts in 0.1 M KOH. References [14,50,51,52,53,54,55,56,57,58,59,60,61,62,63,64,65,66] are cited in the supplementary materials.
