# Supplementary material for: Iron Single Atoms Anchored on Nitrogen-Doped Carbon Matrix/Nanotube Hybrid Supports for Excellent Oxygen Reduction Properties
Source: Nanomaterials (Basel). 2022 May 7;12(9):1593. doi: 10.3390/nano12091593 (PMC9099764; doi:10.3390/nano12091593)
Supplement: Supplementary file 1 [file nanomaterials-12-01593-s001.zip › nanomaterials-1669597-Supplementary.pdf]

Iron Single Atoms Anchored on Nitrogen-Doped Carbon Matrix/Nanotube Hybrid Supports for Excellent Oxygen Reduction Properties

Yining Jia [1], Chunjing Shi [1], Wei Zhang [1], Wei Xia [2], Ming Hu [1], Rong Huang [1,3,*] and Ruijuan Qi [1,*]

[1] Key Laboratory of Polar Materials and Devices (MOE), Department of Electronics Sciences, School of Physics and Electronic Science, East China Normal University, Shanghai 200062, China; 51191213010@stu.ecnu.edu.cn (Y.J.); chunjingshi620@163.com (C.S.); wzhang@ee.ecnu.edu.cn (W.Z.); mhu@phy.ecnu.edu.cn (M.H.)

[2] Shanghai Key Laboratory of Green Chemistry and Chemical Processes, School of Chemistry and Molecular Engineering, East China Normal University, Shanghai 200241, China; xiaweifriend@163.com

[3] Collaborative Innovation Center of Extreme Optics, Shanxi University, Taiyuan 030006, China

* Correspondence: rhuang@ee.ecnu.edu.cn (R.H.); rjqi@ee.ecnu.edu.cn (R.Q.)

Citation: Jia, Y.; Shi, C.; Zhang, W.; Xia, W.; Hu, M.; Huang, R.; Qi, R. Iron Single Atoms Anchored on Nitrogen-Doped Carbon Matrix/Nanotube Hybrid Supports for Excellent Oxygen Reduction Properties. *Nanomaterials* **2022**, *12*, 1593. https://doi.org/10.3390/nano12091593

Academic Editor: Maria E. Davila

Received: 22 March 2022
Accepted: 03 May 2022
Published: 07 May 2022

Publisher's Note: MDPI stays neutral with regard to jurisdictional claims in published maps and institutional affiliations.

Copyright: © 2022 by the authors. Licensee MDPI, Basel, Switzerland. This article is an open access article distributed under the terms and conditions of the Creative Commons Attribution (CC BY) license (https://creativecommons.org/licenses/by/4.0/).

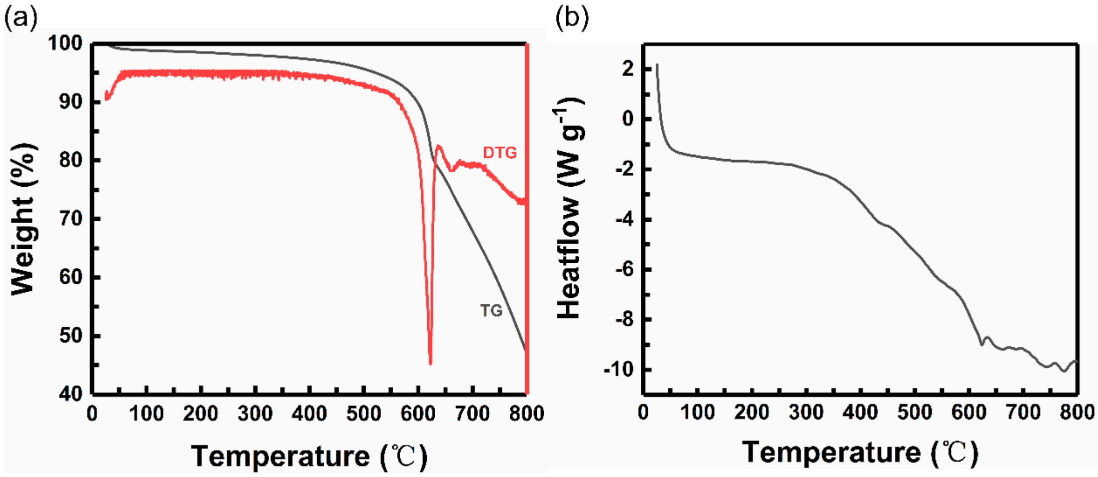

Figure S1. (**a**) TGA and (**b**) DSC curves of $Fe(acac)_3$-0.1@ZIF-8. The weight loss was attributed to the decomposition of ZIF-8 and the release of Zn species. That is, the Zn nodes with a low boiling point of 907°C would evaporate at such high temperatures, leaving the N rich defects.

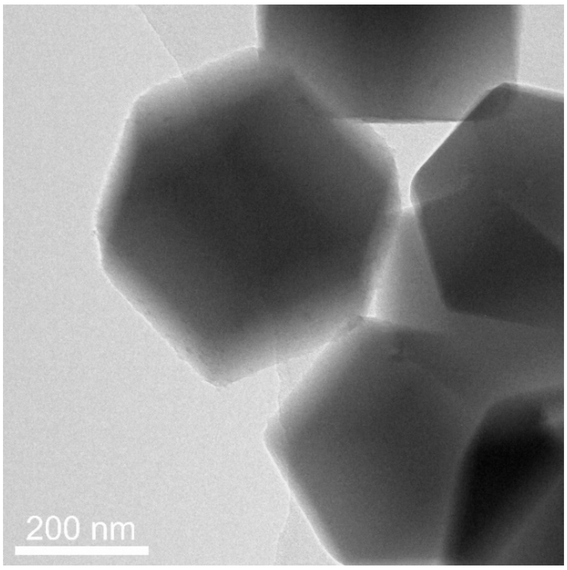

Figure S2. TEM image of $Fe(acac)_3$-0.1@ZIF-8.

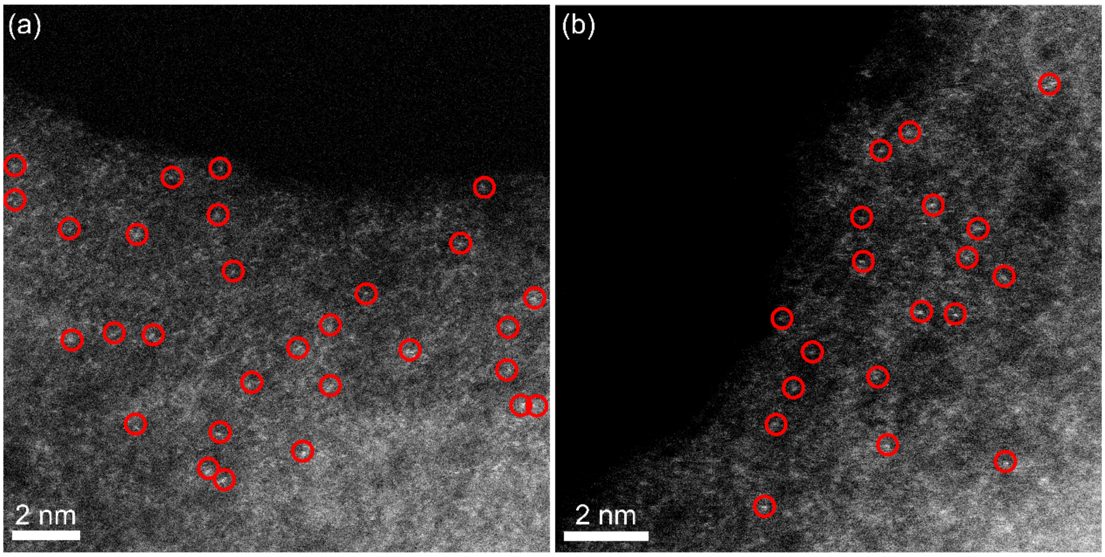

Figure S3. (**a**) and (**b**) are representative HAADF-STEM images of FeSA-NC/CNTs at different areas.

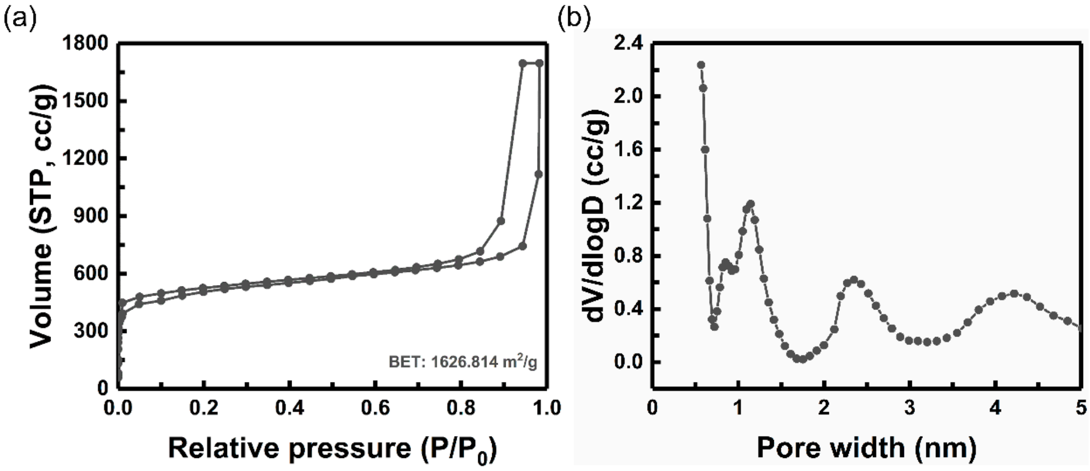

Figure S4. (**a**) N_2 sorption isotherms of FeSA-NC/CNTs. (**b**) Corresponding pore size distribution curve calculated using the DFT methods.

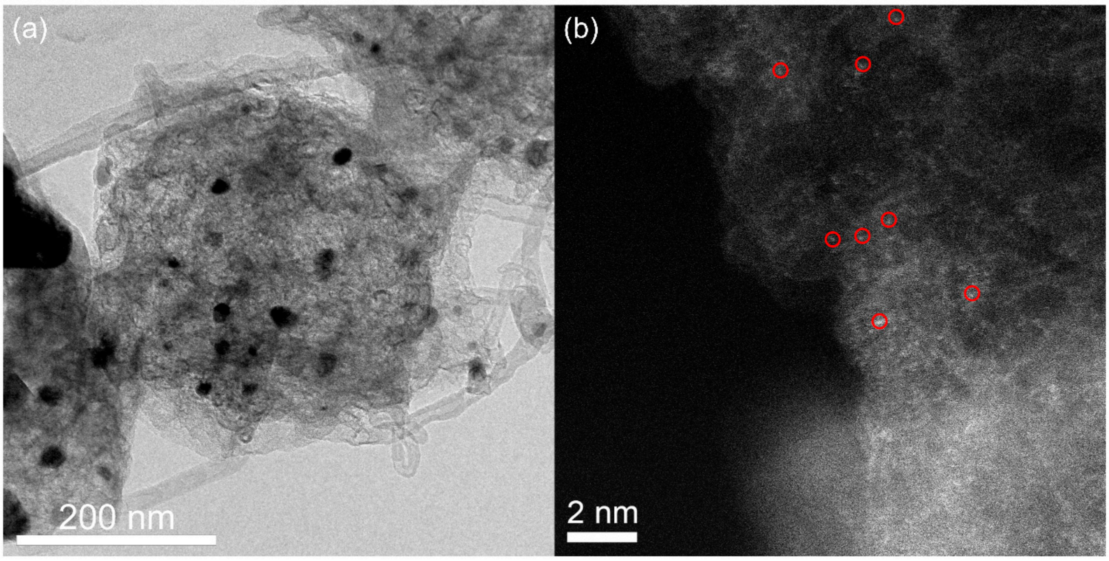

Figure S5. (**a**) TEM and (**b**) HAADF-STEM images of FeNP-NC/CNTs, where typical iron single atoms are marked by red circles.

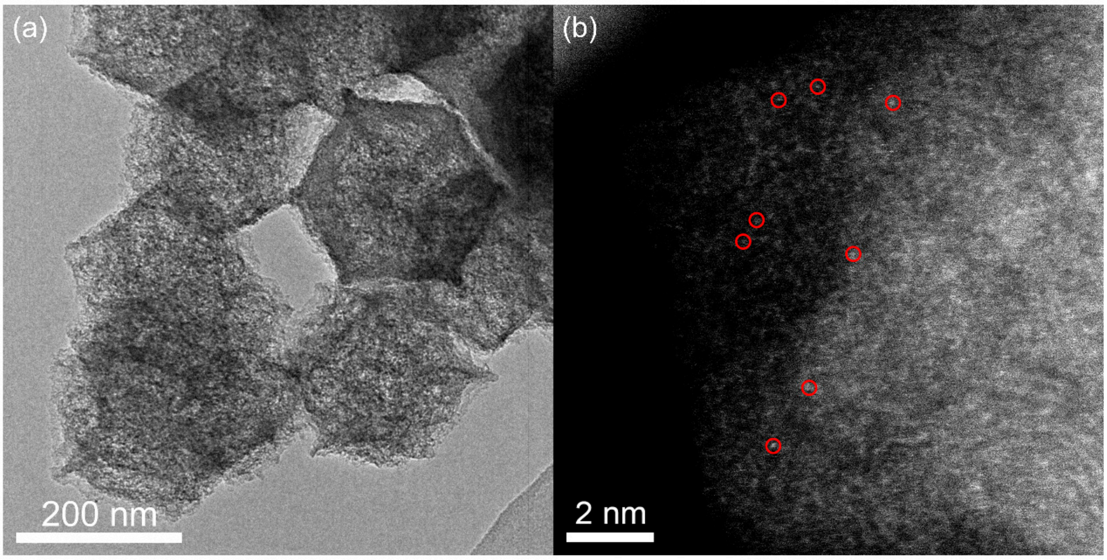

Figure S6. (**a**) TEM and (**b**) HAADF-STEM images of FeSA-NC, where typical iron single atoms are marked by red circles.

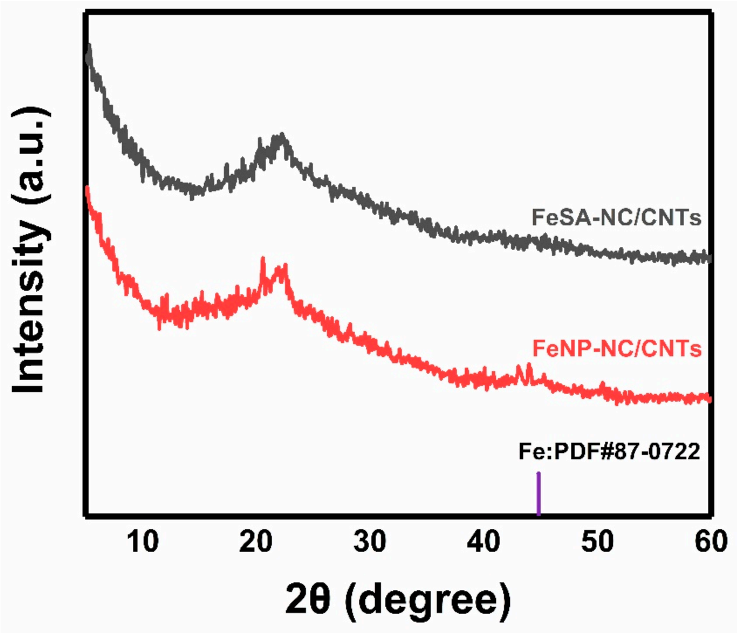

Figure S7. XRD patterns of FeSA-NC/CNTs and FeNP-NC/CNTs.

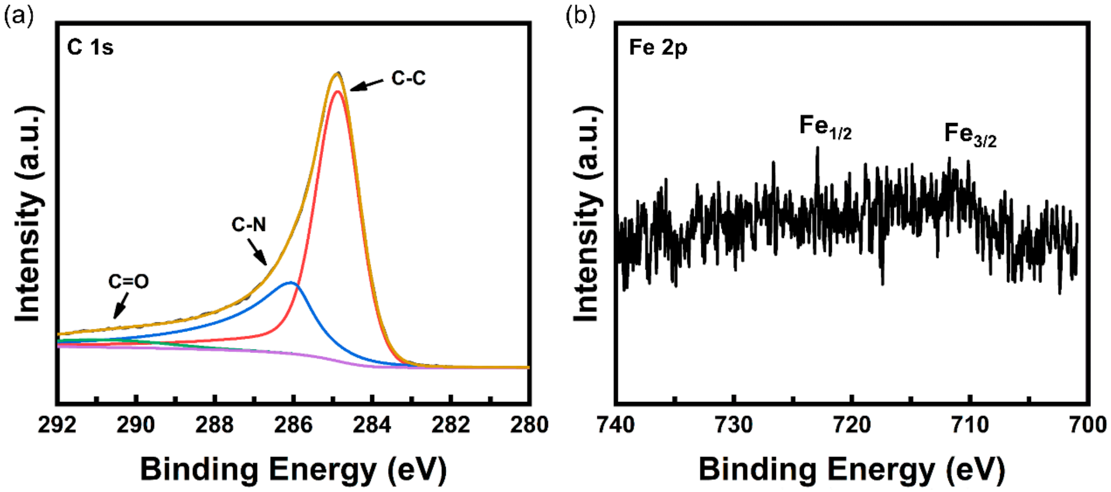

Figure S8. (**a**) High resolution XPS C 1s spectra of FeSA-NC/CNTs. (**b**) High resolution XPS Fe 2p spectra of FeSA-NC/CNTs.

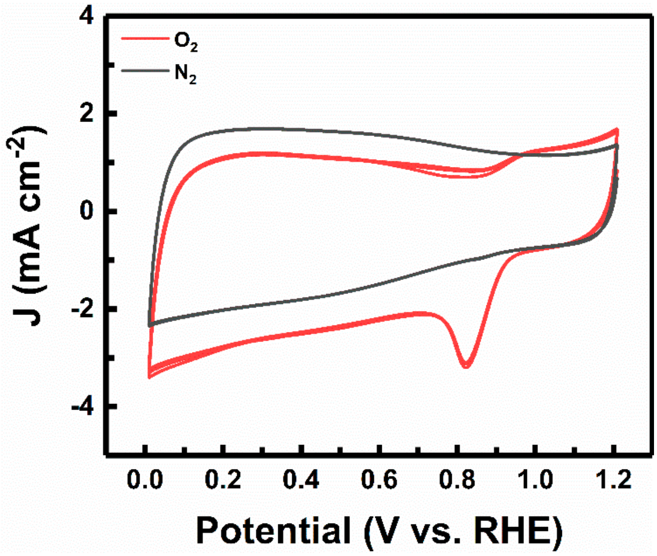

Figure S9. CV curves of FeSA-NC/CNTs in O_2-saturated 0.1 M KOH with a sweep rate of 50 mV/s.

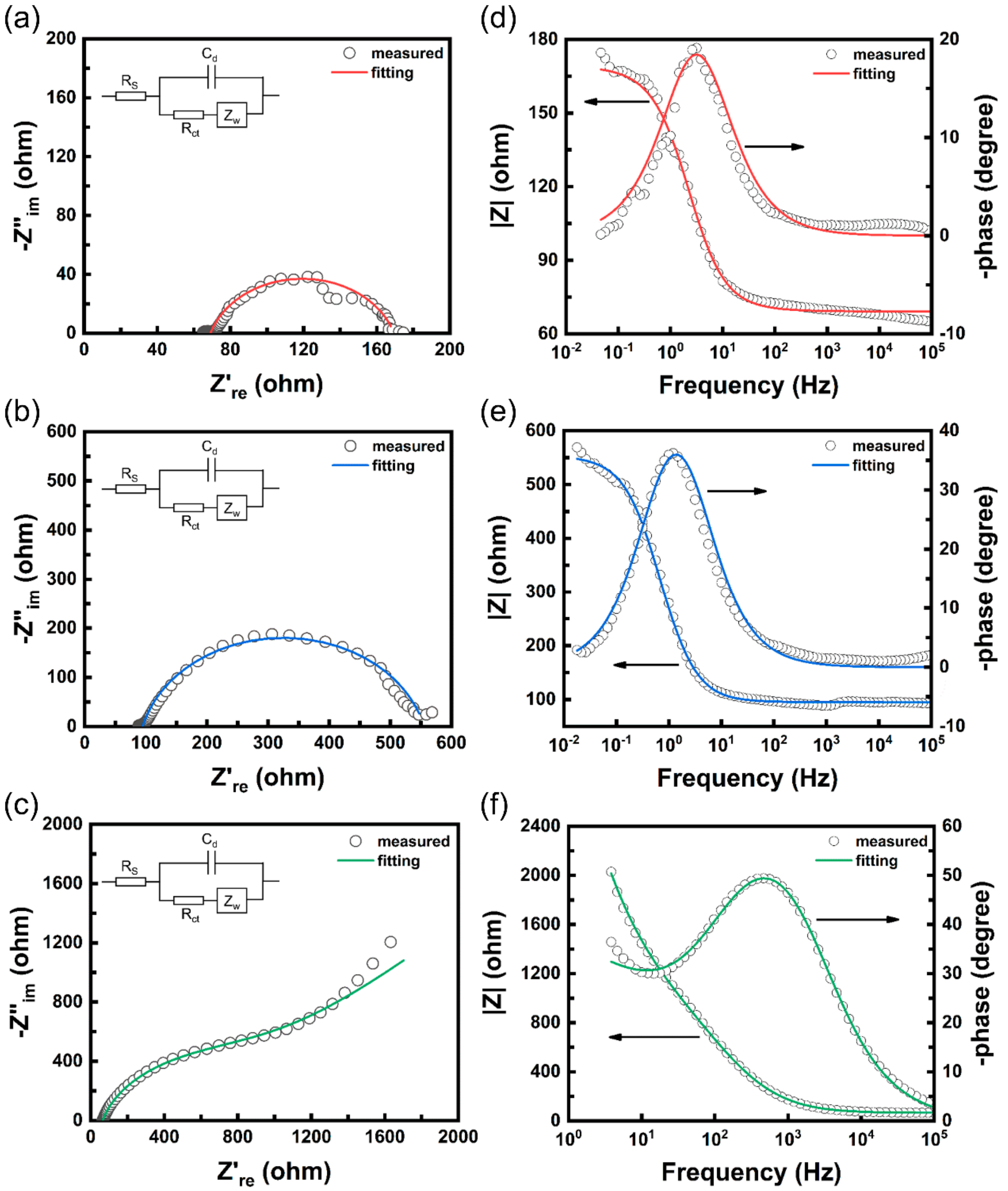

Figure S10. Nyquist plots of (**a**) FeSA-NC/CNTs, (**b**) FeSA-NC and (**c**) FeNP-NC/CNTs, where the inset is the equivalent circuit model for impedance spectra fitting. Bode plots of (**d**) FeSA-NC/CNTs, (**e**) FeSA-NC and (**f**) FeNP-NC/CNTs.

Table S1. Fitting results of Fe foil EXAFS

Sample	Shell	N	R	σ^2	R factor (%)
Fe foil	Fe-Fe	8	2.48	0.008	1.68
	Fe-Fe	6	2.83	0.008	

Fitting range: 3.09≤k (Å^{-1})≤12.15 and 1.34≤R (Å)≤3.00

Table S2. Fitting results of sample-Fe EXAFS

Sample	Shell	N	R	σ^2	R factor (%)
FeSA-NC/CNTs	Fe-N	4.2±0.2	2.01	0.007	0.48

Fitting range: FeSA-NC/CNTs:2.71≤k (Å^{-1})≤8.98 and 1.00≤R (Å)≤2.00

Table S3. Comparison of ORR performance with some reported non-precious catalysts in 0.1 M KOH.

**Catalysts**	**$E_{1/2}$ (V vs. RHE)**	**$E_{1/2}$ relative to Pt/C (mV vs. RHE)**	**J_K at 0.85 V (mV cm^{-2})**	**Tafel slope (mV dec^{-1})**	**Stability**	**Ref.**
FeSA-NC/CNTs	0.86	14	39.3 (@0.8 V)	74.4	1 mV loss after 5000 cycles	This work
SA-Fe-NHPC	0.93	80	6 (@0.7 V)	57.2	1 mV loss after 10000 cycles	[1]
Fe SAs/N-C	0.91	-	-	67	no decay after 10000 cycles	[2]
Fe/N-G-SAC	0.89	-	3.837 (@0.9 V)	50	3 mV loss after 10000 cycles	[3]
Fe SAC-MIL101-1000	0.94	50	37.14 (@0.9 V)	48.8	a current retention of 94.3% at 0.7 V after 40 000 s	[4]
Fe-N_x-C	0.91	90	14.7	69	a current retention of 99% at 0.3 V	[5]

					after 15 h	
Fe-SA/Meso-C	0.926	-	92.5	60.8	6 mV loss after 5000 cycles	[6]
Fe_{SA}/NSC	0.91	40	19.83	55.9	3 mV loss after 5000 cycles	[7]
Fe-SA/NCS	0.91	60	26.65	53.6	a current retention of 95% after 20 h	[8]
Fe-ISAS/CN	0.861	21	5.74	78	4 mV loss after 5000 cycles	[9]
Fe-N-C/N-OMC	0.93	70	87.4	75	5 mV loss after 5000 cycles	[10]
Co/S,N-C	0.84	0	-	76	no decay after 3000 cycles	[11]
Co-SAC/NC	0.884	28	3.48 (@0.9 V)	-	a current retention of 90% after 30000 s	[12]
S-Co/N/C	0.86	-	-	-	a current retention of 94.9% after 100 h	[13]
Co-N/ZIF-2	0.861	42	26 (@0.8 V)	37.9	a current retention of 90% at 0.861 V after 2 h	[14]
Co-SAs@NHOPC	0.851	19	-	51	no decay after 5000 cycles	[15]
Mn-N-C	0.944	-	96.96	64.24	no decay after 5000 cycles	[16]
Ca-N, O/C	0.90	30	-	-	a current retention of 83.2% at 0.7 V after 28 h	[17]
Re SAC	0.72	-80	-	-	a current retention of 80.3% at 0.5 V	[18]

	after 80000 s

Reference:

1. Chen, G.; Liu, P.; Liao, Z.; Sun, F.; He, Y.; Zhong, H.; Zhang, T.; Zschech, E.; Chen, M.; Wu, G. Zinc-mediated template synthesis of Fe-N-C electrocatalysts with densely accessible Fe-Nx active sites for efficient oxygen reduction. *Advanced Materials* **2020**, *32*, 1907399.
2. Ge, X.; Su, G.; Che, W.; Yang, J.; Zhou, X.; Wang, Z.; Qu, Y.; Yao, T.; Liu, W.; Wu, Y. Atomic filtration by graphene oxide membranes to access atomically dispersed single atom catalysts. *ACS Catalysis* **2020**, *10*, 10468-10475.
3. Xiao, M.; Xing, Z.; Jin, Z.; Liu, C.; Ge, J.; Zhu, J.; Wang, Y.; Zhao, X.; Chen, Z. Preferentially engineering FeN4 edge sites onto graphitic nanosheets for highly active and durable oxygen electrocatalysis in rechargeable Zn–air batteries. *Advanced Materials* **2020**, *32*, 2004900.
4. Xie, X.; Peng, L.; Yang, H.; Waterhouse, G.I.; Shang, L.; Zhang, T. MIL-101-Derived Mesoporous Carbon Supporting Highly Exposed Fe Single-Atom Sites as Efficient Oxygen Reduction Reaction Catalysts. *Advanced Materials* **2021**, *33*, 2101038.
5. Han, J.; Meng, X.; Lu, L.; Bian, J.; Li, Z.; Sun, C. Single-atom Fe-Nx-C as an efficient electrocatalyst for zinc–air batteries. *Advanced Functional Materials* **2019**, *29*, 1808872.
6. Wang, X.; Zhu, H.; Yang, C.; Lu, J.; Zheng, L.; Liang, H.-P. Mesoporous carbon promoting the efficiency and stability of single atomic electrocatalysts for oxygen reduction reaction. *Carbon* **2022**, *191*, 393-402.
7. Liu, X.; Zhai, X.; Sheng, W.; Tu, J.; Zhao, Z.; Shi, Y.; Xu, C.; Ge, G.; Jia, X. Isolated single iron atoms anchored on a N, S-codoped hierarchically ordered porous carbon framework for highly efficient oxygen reduction. *Journal of Materials Chemistry A* **2021**, *9*, 10110-10119.
8. Wang, X.; Yang, C.; Wang, X.; Zhu, H.; Cao, L.; Chen, A.; Gu, L.; Zhang, Q.; Zheng, L.; Liang, H.-P. Green Synthesis of a Highly Efficient and Stable Single-Atom Iron Catalyst Anchored on Nitrogen-Doped Carbon Nanorods for the Oxygen Reduction Reaction. *ACS Sustainable Chemistry & Engineering* **2020**, *9*, 137-146.
9. Wei, S.; Wang, Y.; Chen, W.; Li, Z.; Cheong, W.-C.; Zhang, Q.; Gong, Y.; Gu, L.; Chen, C.; Wang, D. Atomically dispersed Fe atoms anchored on COF-derived N-doped carbon nanospheres as efficient multi-functional catalysts. *Chemical science* **2020**, *11*, 786-790.
10. Han, J.; Bao, H.; Wang, J.-Q.; Zheng, L.; Sun, S.; Wang, Z.L.; Sun, C. 3D N-doped ordered mesoporous carbon supported single-atom Fe-NC catalysts with superior performance for oxygen reduction reaction and zinc-air battery. *Applied Catalysis B: Environmental* **2021**, *280*, 119411.
11. Wang, Z.; Shang, N.; Wang, W.; Gao, S.; Zhang, S.; Gao, W.; Cheng, X.; Wang, C. Atomically dispersed Co anchored on S, N-riched carbon for efficient oxygen reduction and Zn-air battery. *Journal of Alloys and Compounds* **2022**, *899*, 163225.
12. Rao, P.; Luo, J.; Wu, D.; Li, J.; Chen, Q.; Deng, P.; Shen, Y.; Tian, X. Isolated Co Atoms

Anchored on Defective Nitrogen-doped Carbon Graphene as Efficient Oxygen Reduction Reaction Electrocatalysts. *Energy & Environmental Materials*.

13. Sun, T.; Zang, W.; Yan, H.; Li, J.; Zhang, Z.; Bu, Y.; Chen, W.; Wang, J.; Lu, J.; Su, C. Engineering the coordination environment of single cobalt atoms for efficient oxygen reduction and hydrogen evolution reactions. *ACS Catalysis* **2021**, *11*, 4498-4509.
14. Wu, H.; Wu, J.; Li, Y.; Li, W.; Zhai, J.; Jiang, Q.; Xu, X.; Gao, Y. Enhanced oxygen reduction with carbon-polyhedron-supported discrete cobalt-nitrogen sites for Zn-air batteries. *Chemical Engineering Journal* **2022**, *431*, 134084.
15. Guo, Y.; Liu, F.; Feng, L.; Wang, X.; Zhang, X.; Liang, J. Single Co atoms anchored on nitrogen-doped hierarchically ordered porous carbon for selective hydrogenation of quinolines and efficient oxygen reduction. *Chemical Engineering Journal* **2022**, *429*, 132150.
16. Kong, Z.; Liu, T.; Hou, K.; Guan, L. Atomically dispersed Mn-N4 electrocatalyst with high oxygen reduction reaction catalytic activity from metal organic framework ZIF-8 by minimal water assisted mechanochemical synthesis. *Journal of Materials Chemistry A* **2022**.
17. Lin, Z.; Huang, H.; Cheng, L.; Hu, W.; Xu, P.; Yang, Y.; Li, J.; Gao, F.; Yang, K.; Liu, S. Tuning the p-Orbital Electron Structure of s-Block Metal Ca Enables a High-Performance Electrocatalyst for Oxygen Reduction. *Advanced Materials* **2021**, *33*, 2107103.
18. Zhang, X.; Shang, L.; Yang, Z.; Zhang, T. A Rhenium Single-Atom Catalyst for the Electrocatalytic Oxygen Reduction Reaction. *ChemPlusChem* **2021**, *86*, 1635-1639.
